# Untreated *Chlorella homosphaera* biomass allows for high rates of cell wall glucan enzymatic hydrolysis when using exoglucanase-free cellulases

**DOI:** 10.1186/s13068-015-0215-1

**Published:** 2015-02-19

**Authors:** Marcoaurélio Almenara Rodrigues, Ricardo Sposina Sobral Teixeira, Viridiana Santana Ferreira-Leitão, Elba Pinto da Silva Bon

**Affiliations:** Federal University of Rio de Janeiro, Institute of Chemistry, Department of Biochemistry, Applied Photosynthesis Laboratory, Athos Avenida da Silveria Ramos, 149-Technology Centre, Block A, Room 532, University City, Rio de Janeiro, RJ 21941-909 Brazil; National Institute of Technology - Ministry of Science, Technology and Innovation, Biocatalysis Laboratory, 20081-312 Rio de Janeiro, RJ Brazil; Federal University of Rio de Janeiro, Institute of Chemistry, Department of Biochemistry, Enzyme Technology Laboratory, 21941-909 Rio de Janeiro, RJ Brazil

**Keywords:** *Chlorella homosphaera*, Chlorophyte cell wall crystallinity, *Acremonium cellulolyticus*, *Trichoderma reesei* and *Aspergillus awamori* cellulases, Microalgae biomass enzymatic hydrolysis, Microalgae glucose sugar syrups

## Abstract

**Background:**

Chlorophyte microalgae have a cell wall containing a large quantity of cellulose I_α_ with a triclinic unit cell hydrogen-bonding pattern that is more susceptible to hydrolysis than that of the cellulose I_β_ polymorphic form that is predominant in higher plants. This study addressed the enzymatic hydrolysis of untreated *Chlorella homosphaera* biomass using selected enzyme preparations, aiming to identify the relevant activity profile for the microalgae cellulose hydrolysis. Enzymes from *Acremonium cellulolyticus*, which secretes a complete pool of cellulases plus β-glucosidase; *Trichoderma reesei*, which secretes a complete pool of cellulases with low β-glucosidase; *Aspergillus awamori*, which secretes endoglucanases and β-glucosidase; blends of *T. reesei*-*A. awamori* or *A. awamori*-*A. cellulolyticus* enzymes; and a purified *A. awamori* β-glucosidase were evaluated.

**Results:**

The highest initial glucan hydrolysis rate of 140.3 mg/g/h was observed for *A. awamori* enzymes with high β-glucosidase, low endoglucanase, and negligible cellobiohydrolase activities. The initial hydrolysis rates when using *A. cellulolyticus* or *T. reesei* enzymes were significantly lower, whereas the results for the *T. reesei*-*A. awamori* and *A. awamori*-*A. cellulolyticus* blends were similar to that for the *A. awamori* enzymes. Thus, the hydrolysis of *C. homosphaera* cellulose was performed exclusively by the endoglucanase and β-glucosidase activities. X-ray diffraction data showing negligible cellulose crystallinity for untreated *C. homosphaera* biomass corroborate these findings. The *A. awamori*-*A. cellulolyticus* blend showed the highest initial polysaccharide hydrolysis rate of 185.6 mg/g/h, as measured by glucose equivalent, in addition to the highest predicted maximum glucan hydrolysis yield of 47% of total glucose (*w*/*w*). *T. reesei* enzymes showed the lowest predicted maximum glucan hydrolysis yield of 25% (*w*/*w*), whereas the maximum yields of approximately 31% were observed for the other enzyme preparations. The hydrolysis yields were proportional to the enzyme β-glucosidase load, indicating that the endoglucanase load was not rate-limiting.

**Conclusions:**

High rates of enzymatic hydrolysis were achieved for untreated *C. homosphaera* biomass with enzymes containing endoglucanase and β-glucosidase activities and devoid of cellobiohydrolase activity. These findings simplify the complexity of the enzyme pools required for the enzymatic hydrolysis of microalgal biomass decreasing the enzyme cost for the production of microalgae-derived glucose syrups.

**Electronic supplementary material:**

The online version of this article (doi:10.1186/s13068-015-0215-1) contains supplementary material, which is available to authorized users.

## Background

In the pursuit of a new, renewable, and environmentally friendly feedstock supply, the use of seed plants, energy crops, grasses, and agricultural wastes has raised serious concerns regarding the use of land and water for food versus feedstock for chemicals and fuel production. Alternatively, attention has been turned to the use of algae, in particular microalgae, as feedstock for the production of renewable chemicals and biofuels, as they do not compete for arable land [1] despite some skeptical views [2,3]. Microalgae are versatile photosynthetic organisms that can adapt to and tolerate a variety of environmental conditions and can be cultivated in non-freshwater sources including salt and wastewater [4,5] besides being able to use flue gases as a source of carbon, sulfur, and nitrogen [6]. The calorific values of microalgae can be increased by growing them in tubular bioreactors [7] or in cheaper, low nitrogen media [8] because nitrogen starvation triggers lipid and carbohydrate accumulation [9]. Several products can be obtained from the same algal biomass using an integrated biorefinery concept whereby different biomass processing methods can be sequentially employed, including lipid extraction, algae biomass polysaccharide hydrolysis for sugar syrup production and subsequent fermentation [10], followed by biodigestion or pyrolysis for the production of biodiesel, bioethanol, methane, and syngas [11-13]. Furthermore, milder conditions for microalgae, with the aim of biomass polysaccharide hydrolysis, are necessary for the production of sugar syrups in comparison with lignocellulosic biomass processing [14,15].

The carbohydrates in green algae, primarily cellulose, come chiefly from the cell wall components although depending on the growth conditions in which starch accumulates in the chloroplasts [16]. Early studies on the algal cell wall [17] classified the algae species into three groups according to their cell wall constituents. Group 1 includes chlorophyte algae, in which native cellulose is the major component of the cell walls and is usually highly crystalline. These algae belong to the Cladophorales order. Group 2 includes the chlorophyte algae, which have cell walls that contain a large quantity of mercerized-like cellulose, which is presumably a derivative of native cellulose. This cellulose has a low degree of crystallinity, and the chains are randomly oriented. Most algae fall into this category. Group 3 contains algae for which the walls contain a well-oriented and highly crystalline skeletal substance, which is neither native nor mercerized cellulose, as the major constituent [17]. Three types of cellulose were identified in the algal system as follows: the I_α_-rich, broad microfibril, 0.6 nm-oriented type; the I_β_-dominant flat-ribbon, 0.53 nm-oriented type; and the I_β_-dominant, small, random-oriented type. The first type appears to occur in more primitive organisms than the other types. The three types of algal cellulose correlate well with the arrangements of cellulose synthesizing complexes, i.e., a multiple-row linear type, a consolidated rosette type, and an isolated rosette type, respectively [18,19]. Linear-type terminal complexes are found in algae belonging to the Chlorophyta division whereas the rosette terminal complex is found in algae belonging to the Charophyta division and land plants [18-21]. Cellulose I_α_ differs from I_β_ not only in the hydrogen-bonding pattern, and consequently in the crystalline unit cell [22,23], but also in its stability and susceptibility to hydrolysis. Cellulose I_α_, which possesses a triclinic unit cell, is metastable and converts into the monoclinic form (I_β_) after annealing in dilute alkali at 260°C [24]; the theoretical density of the monoclinic unit cell is slightly greater than that of the triclinic unit cell [25]. These differences in the stability, hydrogen-bonding pattern, and distribution of the two crystalline phases in the microfibril may render cellulose I_α_ more prone to enzymatic hydrolysis, as indicated by FTIR and electron diffraction data [25,26]. The X-ray diffraction patterns of cellulose in plants and algae differ in the presence of well-resolved and narrow peaks, especially at 2*θ*’s of 14° and 16°, which are not common for native cellulose obtained from higher plants [27].

*Chlorella homosphaera* is a microalga that belongs to the Chlorophyta division. The microalgae from this genus divide by autosporulation and therefore possess lytic activity [28-31] that leads to the enzymatic dissolution of their inner cell wall polysaccharides. The *Chlorella* cell wall is formed by an inner and outer layer and possesses unusual polysaccharides other than cellulose and hemicellulose [32,33]. The inner layer is composed of a matrix and a rigid fibrillar structure, shows high cellulose content, and is susceptible to degradation by cellulolytic enzymes [34]. The outer cell wall, which is resistant to treatment with a number of enzymes, presents two types of ultrastructure, one of which is a trilaminar structure with several resistant components including the biopolymer algaenan, a non-hydrolyzable aliphatic biopolymer composed of long-chain, even-carbon-numbered, ω9-unsaturated ω-hydroxy fatty acid monomers that vary in their chain lengths from 30 to 34 carbon atoms. These monomers are intermolecularly ester-linked to form linear chains in which the unsaturated carbons act as the starting position of ether cross-linking [35].

This study evaluates the enzymatic hydrolysis of polysaccharides from untreated *C. homosphaera* biomass with enzyme preparations that were produced by the fungi *Trichoderma reesei*, *Aspergillus awamori*, and *Acremonium cellulolyticus*, in addition to the mixtures of enzymes produced by *T. reesei* and *A. awamori* or *A. cellulolyticus* and *A. awamori.* The focus was to identify the enzyme activities necessary for the efficient hydrolysis of *C. homosphaera* cell wall polysaccharides because each enzyme preparation showed a different enzyme activity profile and load concerning its endoglucanase, cellobiohydrolase, and β-glucosidase content, aside from the differences regarding their xylanolytic enzyme profiles. This work also reports on the initial hydrolysis rates, the effect of the β-glucosidase load on the hydrolysis rates, and the final hydrolysis yields. The XRD crystallinity profile of the *C. homosphaera* cell wall from untreated algal biomass is also presented.

## Results and discussion

### *C. homosphaera* biomass sugar composition

Data for the total sugar content and sugar composition of *C. homosphaera* biomass dry weight for the biomass total hydrolysis by 1 mol/L H_2_SO_4_ followed by high-performance anion exchange chromatography and pulse amperometric detection (HPAEC) analysis is presented in Table 1. *C. homosphaera* biomass possessed a carbohydrate content of 59.7% with a glucose contribution of 53.9%. Considering that amyloglucosidase hydrolysable starch (AHS) contributed to 7% (*w*/*w*) of the total glucose, non-AHS polysaccharides corresponded to 46.1% of the *C. homosphaera* biomass. As such, glucose constituted 90.4% of all carbohydrates present in *C. homosphaera* (85.5% non-AHS polysaccharides) besides the minor contributions of galactose (5.4%), arabinose (2.6%), mannose (1.3%), and xylose (0.5%), which makes this microalga a glucose-rich biomass. This high carbohydrate content is caused by the growth conditions used in this study; after nitrate depletion, within the fifth day of cultivation, the microalga was kept under nitrogen starvation for 15 days before harvest. Nitrogen starvation reportedly triggers the accumulation of carbohydrates, primarily starch, and lipids in both *Chlorella* [9,16,36] and *Chlamydomonas reinhardtii* [37,38], increasing their calorific value [8]. The low AHS content of *C. homosphaera* late stationary grown phase cells found in this study suggests that reserve carbohydrates, which may have accumulated during the exponential phase of growth as suggested by the Lugol’s solution coloration used for cell counting (data not shown), were converted into structural carbohydrates and incorporated into the algal cell wall [37-39] primarily as a glucan type, most likely cellulose. Accordingly, cellulose was the primary polysaccharide present in *Chlorella fusca* [30,31] and *Coelastrum sphaericum* [35]; however, a similar low starch content was found in the chlorophyte *Chlorococcum humicola* [40].

**Table 1:** **The sugar composition determined by the HPAEC-PAD of** ***C. homosphaera*** **after acidic hydrolysis with 1 mol/L H**_2_ **SO**_4_

**Sugar**	**% Biomass**	**% Total sugar**
Glucose	53.9 (5.4)	90.4 (9.0)
Galactose	3.2 (0.2)	5.4 (0.3)
Arabinose	1.6 (0.1)	2.6 (0.2)
Mannose	0.8 (0.1)	1.3 (0.1)
Xylose	0.3 (0.0)	0.5 (0.0)
Total	59.7 (5.6)	100.2

Hydrolysis and HPAEC-PAD analysis conditions are described in the ‘Materials and methods’ section. The numbers shown in the brackets are the standard deviations of the means.

These results resemble those previously reported for the cell wall sugar composition of some Chlorophytes [41-43], for which glucose, galactose, mannose, arabinose, xylose, rhamnose, and sometimes fucose were detected. A similar composition was also found in the TFA-hydrolyzed microalgal biomass of *C. reinhardtii* [37] and mucilage sheaths of *C. sorokiniana*, although sucrose was among the saccharides that were found [44]. Interestingly, glucose and mannose are the monosaccharides that are reportedly found in the rigid fibrillar structure of the inner layer of the cell wall, and all the others were found only in the matrix structure of the inner layer, with glucose as the primary saccharide [42,43]. Because we estimated the sugar composition of the *C. homosphaera* total biomass and not that of the purified cell walls, we expected that non-cell wall polysaccharides such as starch would contribute to the total glucose composition, as already noted; nevertheless, the chlorophyte microalgae *Scenesdesmus obliquus* possesses a cell wall that is enriched with glucose [45].

### Biomass enzymatic hydrolysis

Hydrolysis experiments were performed using enzymes that were produced by selected fungal strains according to their different profiles in relation to cellulase, β-glucosidase, and xylanase activities, as follows: *Acremonium* cellulase (Meiji Seika Co., Japan), a preparation possessing a complex set of biomass-hydrolyzing enzyme activities; exoglucanase (4.3 filter paper unit (FPU)/mL) and endoglucanase (67.1 IU/mL); xylanase (208.0 IU/mL) and β-glucosidase (29.2 IU/mL); a *T. reesei* RUT C-30 preparation containing exoglucanase (1.2 FPU/mL), endoglucanase (37.3 IU/mL), xylanase (228.0 IU/mL), and low β-glucosidase content (1.4 IU/mL); the *A. awamori* 2B.361 U2/1 enzyme was devoid of exoglucanase but exhibited xylanase (117.0 IU/mL), β-glucosidase (8.4 IU/mL), and low endoglucanase (1.9 IU endo-β-1,4 glucanase (CMCase)/mL).

The *A. awamori*-*T. reesei* blend contained exoglucanase (5.3 IU FPU/mL), endoglucanase (20.5 IU/mL), xylanase (202.0 IU/mL), and β-glucosidase (69.5 IU/mL) activities; the supernatants of individual fungal cultures were concentrated by ultrafiltration using a 30-kDa membrane before blending. The *A. awamori*-*A. cellulolyticus* blend possessed exoglucanase (2.0 FPU/mL), endoglucanase (32.5 IU/mL), xylanase (159.7 IU/mL), and β-glucosidase (18.2 IU/mL) activities.

### Sugar analyses

Table 2 shows the sugar composition and yield released from the *C. homosphaera* biomass after 48 h of enzymatic hydrolysis. The overall analysis of the effectiveness of the different enzyme preparations in hydrolyzing the algae cell wall polysaccharides indicates that the *A. awamori*-*A. cellulolyticus* enzyme blend released the highest amounts of glucose and galactose, besides releasing mannose, and was the only preparation that was able to release the pentoses xylose and arabinose and thus able to attack pentose-containing polysaccharides, besides glucan and galactose and/or mannose-containing polysaccharides. The *A. awamori*-*T. reesei* blend was able to hydrolyze glucan aside from the galactose and/or mannose-containing polysaccharides, and all the other enzyme preparations were solely able to hydrolyze glucan and galactose-containing polysaccharides, which were noteworthy in that the *A. cellulolyticus* enzymes released the largest amount of galactose. Partially purified β-glucosidase was solely able to release limited amounts of glucose in comparison with the non-enzymatic control experiments; the small detected galactose release can be considered non-enzymatic when taking into account the data for the non-enzymatic galactose release. No cellobiose was detected in the hydrolysate for all enzyme preparations. Because *A. awamori* can also produce amylases [46], one could argue that the high glucose content found in the hydrolysates of *A. awamori* and the *T. reesei*-*A. awamori* blend might result from starch hydrolysis. To test this hypothesis, 50 mg/mL starch solution was incubated under the same conditions used for algae biomass hydrolysis for each enzyme preparation; however, all preparations were unable to hydrolyze starch (data not shown). Because the *C. homosphaera* starch content was not converted into glucose, all the measured glucose was solely released from the cell wall polysaccharides.

**Table 2:** **The sugar composition of hydrolyzed** ***C. homosphaera*** **biomass after 48 h of enzymatic treatment**

**Enzyme preparation**	**Glucose**		**Galactose**		**Mannose**		**Total yield (%)**
	**g/L**	**Yield (%)**	**g/L**	**Yield (%)**	**g/L**	**Yield (%)**	
*T. reesei*	6.6 (0.0)	23.4 (0.1)	0.2 (0.0)	0.5 (0.0)	0.0	0.0	24.0 (0.1)
*A. cellulolyticus*	9.9 (0.4)	35.0 (1.3)	0.5 (0.0)	1.8 (0.0)	0.0	0.0	36.7 (1.3)
*A. awamori*	10.0 (0.2)	35.4 (0.6)	0.3 (0.0)	1.2 (0.0)	0.0	0.0	35.4 (0.6)
*T. reesei*-*A. awamori*	9.4 (0.2)	33.1 (0.7)	0.1 (0.0)	0.4 (0.0)	0.1 (0.0)	0.1 (0.0)	33.5 (0.7)
*A. cellulolyticus*-*A. awamori* ^a^	11.4 (1.2)	40.2 (4.2)	0.6 (0.0)	2.0 (0.1)	0.0 (0.0)	0.1 (0.0)	42.4 (4.3)
β-Glucosidase	2.1 (0.1)	7.6 (0.3)	0.1 (0.0)	0.3 (0.0)	0.0	0.0	7.9 (0.3)
Control	1.2 (0.1)	4.2 (0.3)	0.1 (0.0)	0.4 (0.0)	0.0	0.0	4.6 (0.4)

The results are shown as the sugar concentration (g/L) and hydrolysis yield in terms of the total sugar concentration (%). The hydrolysis and HPAEC-PAD analysis conditions are described in the ‘Materials and methods’ section. As a control, the sugar released from the biomass that was incubated without any enzyme preparation is also shown. Numbers shown in brackets are standard deviations of the means.

^a^Xylose and arabinose at a low concentration (0.03 g/L; 0.12% yield) were released solely for the hydrolysis performed by the *A. cellulolyticus*-*A. awamori* enzyme blend.

Glucose and mannose are reportedly found in the rigid fibrillar structure of the inner layer of the *C. homosphaera* cell wall, and galactose, arabinose, and xylose are present in the matrix of the cell wall inner layer [42,43]. Thus, the absence of mannose in all enzyme preparations but its presence in the *A. awamori*-*A. cellulolyticus* and *A. awamori*-*T. reesei* blends might indicate that these enzyme preparations are able to hydrolyze only the glucan and galactose-containing polysaccharides in the matrix of the inner cell wall. However, the presence of mannose in the hydrolysates resulting from the use of *A. awamori*-*A. cellulolyticus* and *A. awamori*-*T. reesei* blends indicate that these preparations were able to attack the rigid fibrillar structure of the inner cell wall layer. Moreover, because xylose and arabinose, in addition to glucose, galactose, and mannose, were detected in the hydrolysates resulting from the use of the *A. awamori*-*A. cellulolyticus* blend, this preparation was also able to attack different glycosidic bonds or a different structural polysaccharide. The use of enzyme blends showed the synergistic effect of the enzymes produced by these two fungi. The importance of hemicellulases in the polysaccharide hydrolysis in the algae inner layer cell wall was not assessed in this study. However, even when considering that substances other than glucose monosaccharides represent less than 10% of the total algae biomass sugar, key glucosidic bonds involving galactose, arabinose, mannose, and xylose may hinder high-yield glucan hydrolysis. In this context, it is noteworthy that the best performing enzyme preparation, i.e., the *A. awamori*-*A. cellulolyticus* blend that resulted in the highest glucose hydrolysis yield, also released the highest amount of galactose, besides releasing mannose, arabinose, and xylose, most likely because of the action of the hemicellulose-degrading enzyme complex.

Nevertheless, interesting results were obtained in this study with regard to the selective hydrolysis of structures in the algae inner cell wall, and conclusive data regarding the specificity of the selected enzyme pool towards the structural polysaccharides could only be gathered from hydrolysis experiments that were performed with purified components of the cell wall. Those results would be compared to sequentially TFA and H_2_SO_4_-hydrolyzed cell wall components because TFA hydrolyzes only the matrix of the inner cell wall and H_2_SO_4_ hydrolyzes both the matrix and the inner cell wall fibrillar structure [47]. This question will be a matter of further investigation.

**Figure 1:**
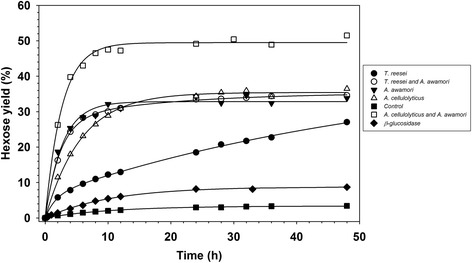
**A time course for the release of total reducing sugars from the** ***C. homosphaera*** **biomass (50 mg d.w./mL) in 50 mM citrate buffer, pH 4.8, at 50°** **C.** The biomass was hydrolyzed using the enzyme preparations from *T. reesei* (filled circles), *A. cellulolyticus* (open triangles), *A. awamori* (inverted filled triangles), the *T. reesei*-*A. awamori* blend (open circles), the *A. cellulolyticus*-*A. awamori* blend (open squares), and partially purified β-glucosidase (diamonds). Biomass suspended in buffer without enzyme preparation was used as a control (filled squares). The data were fitted into an exponential function as described in the ‘Materials and methods’ section. Standard deviation was less than 10% of the mean value, and bars were omitted for clarity.

**Figure 2:**
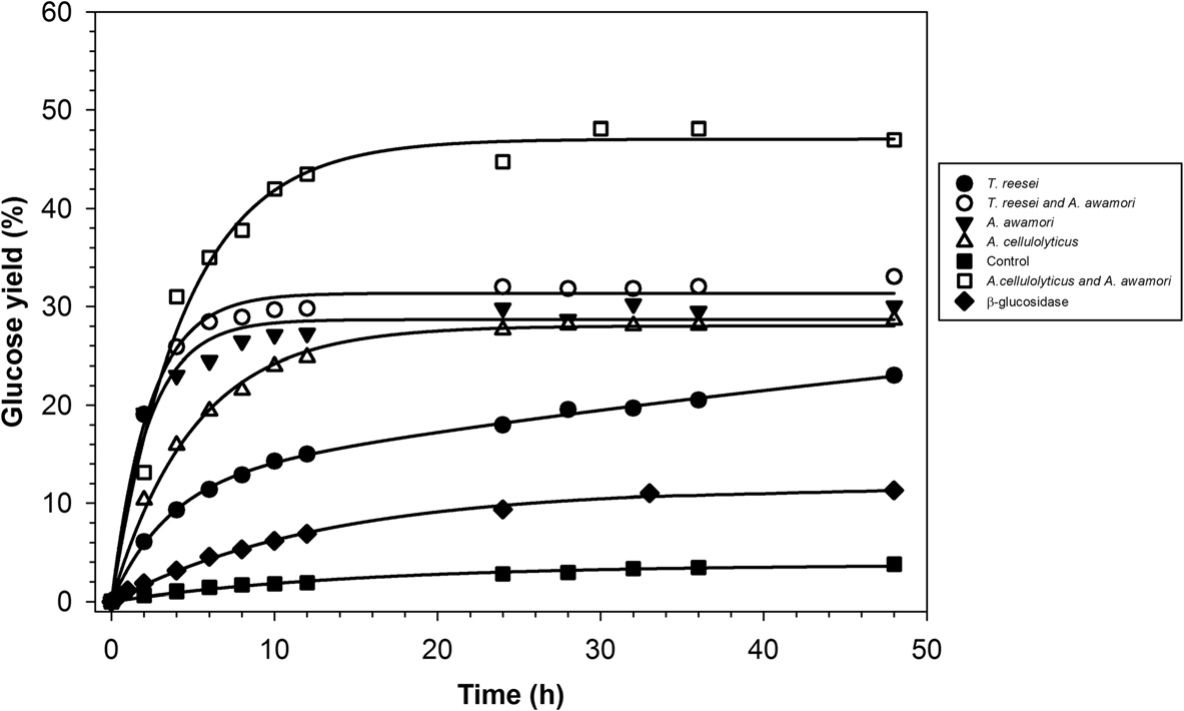
**The time course for the release of glucose from** ***C. homosphaera*** **biomass (50 mg d.w./mL) in 50 mM citrate buffer, pH 4.8, at 50°** **C** **.** The biomass was hydrolyzed using the enzyme preparations from *T. reesei* (filled circles), *A. cellulolyticus* (open triangles), *A. awamori* (inverted filled triangles), the *T. reesei*-*A. awamori* blend (open circles), the *A. cellulolyticus*-*A. awamori* blend (open squares), and partially purified β-glucosidase (diamonds). Biomass suspended in buffer without enzyme preparation was used as the control (filled squares). The data were fitted into an exponential function as described in the ‘Materials and methods’ section. Standard deviation was less than 10% of the mean value and bars were omitted for clarity.

These results are consistent with the time course of algae biomass hydrolysis shown in Figures 1 and 2. The glucose and total reducing sugar yields curves are very similar when the biomass was hydrolyzed with the enzymes of *T. reesei*, *A. awamori*, or a mixture of both. However, when the enzymes of *A. cellulolyticus* or the *A. awamori*-*A. cellulolyticus* blend were used, the amount of released glucose was noticeably lower than that of the total reduced sugar, suggesting that non-glucan-type polysaccharides were hydrolyzed.

### *C. homosphaera* biomass enzymatic hydrolysis

Figures 1 and 2 present time course profiles for the release yield of total reducing sugars and of glucose, in terms of total sugar and total glucose, respectively, on the enzymatic hydrolysis of *C. homosphaera* biomass. In Figure 1, curves for the *A. cellulolyticus*, *A. awamori*, *A. cellulolyticus*-*A. awamori* blend, partially purified β-glucosidase, and the control were best fitted into a mono-exponential model, and the curves for *T. reesei* and the *T. reesei*-*A. awamori* blend were best fitted into a double exponential model. In Figure 2, the curve for *T. reesei* was best fitted into a double exponential model, and the others were best fitted into a mono-exponential model. Figure 1 shows that the initial rates and final yields for the release of reducing sugars for the *A. cellulolyticus*-*A. awamori* blend were the highest followed by *A. awamori* and *T. reesei*-*A. awamori* blend enzyme preparations, which were comparable to one another, plateauing at a 10-h reaction time. The enzymes from *A. cellulolyticus* exhibited a lower initial rate, plateauing with comparable hydrolysis yields at an 18-h reaction time. Enzymes from *T. reesei* showed the smallest initial rate and did not reach the yield plateau within the time frame of the hydrolysis reaction. The partially purified β-glucosidase, which is devoid of cellobiohydrolase, endoglucanase, and xylanase activities, showed a discrete ability to hydrolyze the *C. homosphaera* biomass with a low initial rate and maximum yield, indicating that the β-glucosidase excreted by *A. awamori* was able to attack the algae biomass glucan in addition to its well-known action on cellobiose and short chain oligosaccharides [48]. However, the results showed that the synergistic action of endoglucanases and β-glucosidase is essential to attain higher hydrolysis yields. The *C. homosphaera* biomass also showed a small amount of sugar release unrelated to the enzymatic action (control) for up to 10 h of incubation, possibly because of the reaction conditions, including the pH and temperature, or because of the residual action of native autolytic enzymes. It is well established that chlorophytes reproduce by autospore formation, in which daughter cells are enclosed inside the mother cell wall; autolytic enzymes are used to degrade the mother cell wall and release the daughter cells, which then may use the hydrolyzed monosaccharides as carbon and energy sources [29-31,34]. Figure 2 shows the algae biomass hydrolysis pattern, including the initial rates and final yields for glucose release for all enzyme preparations. The general enzyme preparation performances were quite similar, as expected considering that glucan is the most abundant component of the *C. homosphaera* cell wall, except for the fact that the initial rate of glucose release for the *A. awamori*-*A. cellulolyticus* blend was considerably smaller than the release of total reducing sugar, which was consistent with the results shown in Table 2.

Table 3 summarizes the data for the predicted kinetic parameters regarding initial hydrolysis rates and maximum hydrolysis yields. The data are related to the total sugar (hexose equivalents) and total glucose (glucose) hydrolysis yields. The initial hydrolysis rate of *A. awamori* preparation, when considering hexose equivalents, was 114.3 mg hexose/g total sugar/h, and the rate for the *T. reesei*-*A. awamori* blend was 115.8 mg hexose/g total sugar/h. Enzymes from *A. cellulolyticus* showed a lower initial velocity of 58.8 mg hexose/g total sugar/h); nevertheless, the maximum predicted hydrolysis yield of 336.2 mg/g was similar to that for *A. awamori* (311.7 mg/g) and for the *T. reesei*-*A. awamori* blends (335.4 mg/g). Enzymes from *T. reesei* showed the smallest initial rate (27.5 mg hexose/g sugar/h); because the hydrolysis plateau was not reached within the time frame of the experiment and the predicted maximum value by the model was unreliable, the mean experimental result (±standard deviation) at 48 h of incubation was used as the data for the hydrolysis yield of 256.8 mg/g. The highest initial rate and maximum yield were attained with the *A. awamori*-*A. cellulolyticus* blend with an initial rate and a final yield that were considerably superior to all the other enzymes preparations (185.6 mg/g/h and 494.6 mg/g, respectively), suggesting that the enzymes of these fungi act synergistically in hydrolyzing the polysaccharides that each enzyme preparation cannot hydrolyze by itself as already shown by the HPAEC-PAD. The partially purified β-glucosidase showed a low initial rate and maximum yield and the biomass incubated without enzymes released some sugar at a very low initial rate and yield.

**Table 3:** **Predicted kinetic parameters for** ***C. homosphaera*** **enzymatic hydrolysis yields**

**Enzyme preparation**	**Predicted parameters**					
	**Hexose equivalents (SE)**			**Glucose (SE)**		
	***V***_0_ **(mg/g/h)**	***P***_max_ **(mg/g)**	***t*** **½ (h)**	***V***_0_ **(mg/g/h)**	***P***_max_ **(mg/g)**	***t*** **½ (h)**
*T. reesei* ^a^	27.5 (6.3)	256.8 (38.9)	12.9	33.7 (4.3)	255.56 (40.2)	5.9
*A. cellulolyticus*	58.8 (2.1)	336.2 (3.2)	4.0	61.82 (2.7)	311.1 (3.5)	3.5
*A. awamori*	114.3 (9.0)	311.7 (9.0)	1.9	140.3 (16.9)	318.2 (6.3)	1.6
*T. reesei* and *A. awamori*	115.8 (16.1)	335.4 (27.3)	2.2	149.8 (29.2)	348.0 (11.3)	1.6
*A. cellulolyticus* and *A. awamori*	185.6 (16.7)	494.6 (8.6)	1.8	103.5 (7.9)	470.8 (9.3)	3.2
β-Glucosidase	8.4 (0.4)	88.0 (1.9)	7.3	8.9 (0.5)	116.0 (2.6)	9.0
Control	3.1 (0.1)	33.6 (0.5)	7.4	2.5 (0.3)	37.3 (1.5)	10.4

The data were fitted into an exponential model as described in the materials and methods, and the parameters were estimated as follows: the *V*_0_ was estimated by the limit of the first derivative of the fitting function when time → 0, *P*_max_ was estimated by the limit of the fitting function when time → ∞, *t*½ was estimated using the curve fitting model by calculating the predicted *P*_max_/2. The number in brackets accounts for the standard error of the estimation.

^a^Experimental results were used for the calculations because the parameters predicted by the fitting function showed very large standard errors. The *V*_0_ was calculated using the first experimental result, i.e., the hydrolysis yield after two hours of incubation. The *P*_max_ was estimated using the 48 h hydrolysis yield. The *t*½ was estimated by calculating the *P*_max_/2 and estimating the corresponding time using the fitting function.

Following the same trend, the enzyme pool of *A. awamori* and the blends of *A. awamori*-*T. reesei* and *A. awamori*-*A. cellulolyticus* took approximately 2 h to accomplish 50% of the maximum hydrolysis yield (*t*½). *A. cellulolyticus* enzymes took 4 h and *T. reesei* enzymes took 13 h to achieve 50% of the 48 h hydrolytic value (Table 3). The predicted kinetic parameters for the enzymatic hydrolysis results when using all enzyme preparations, and taking into account the glucose concentration measurements, were similar to those observed for the measurement of the total reducing sugars, as previously discussed, except for the *A. awamori*-*A. cellulolyticus* blend, which showed a similar initial rate for glucose release when compared with the enzyme preparations of *A. awamori* and the *A. awamori*-*T. reesei* blend. Because the glucose yield was considerably higher (470.8 mg glucose/g sugar), the *t*½ was larger for the *A. awamori*-*A. cellulolyticus* blend (Figures 1 and 2 and Table 3). The initial velocities found for *T. reesei* and *A. cellulolyticus* enzymes are close to those previously reported by Harun and Danquah [40] for *Chlorococum humicola* when the same enzyme load was applied. Nevertheless, those found for *A. awamori* and *A. awamori*-*T. reesei* were 1.5 times higher and those for the *A. awamori*-*A. cellulolyticus* blend were 2.4 higher. Moreover, with the exception of the *T. reesei* enzyme pool, we used the Marquardt non-linear regression method, which is a much more reliable procedure for estimating the initial velocity (Table 3). These results suggest that the *A. awamori* enzyme pools efficiently hydrolyze *C. homosphaera* biomass, and the results observed for the *A. awamori*-*T. reesei* blend resulted predominantly from the contribution of the *A. awamori* enzyme pool. The *A. awamori*-*A. cellulolyticus* blend hydrolyzes the *C. homosphaera* biomass synergistically to attain a high rate and yield for hydrolyses.

The maximum hydrolytic yield found for all enzyme pools were either similar [40] or lower, sometimes considerably lower (near 50%), than those previously reported [16]. However, in reports in which a higher hydrolytic yield was found, the algae biomass showed a fourfold higher starch content [16], and the material was subjected to severe treatment beforehand, such as sonication, disc milling, or hydrothermal, acid, or alkali pre-treatment before enzymatic hydrolysis [14,16,38,40,45], which may have disrupted the structure of the outer cell wall. In the present work, the room temperature dried algal biomass was only manually ground, which is a very mild procedure for disrupting the algae cell wall; therefore, the enzymatic hydrolysis results reported herein are quite promising.

The maximum yield was near 50 mg of reducing sugar/g total sugar, indicating that approximately 50% of the algae polysaccharides were not accessed by either of the studied enzyme preparations. Considering the structure of the whole cell wall and that the outer cell wall is resistant to treatments with a number of enzymes, we could speculate that only the inner cell wall layer polysaccharides from either the rigid fibrillar structure or the matrix were selectively or entirely hydrolyzed by the studied enzyme preparations. As such, the residual non-hydrolyzed polysaccharide fraction, from the higher yield hydrolysis experiments, would be a component of the outer cell wall. In reports in which the starch content was similar to ours, a comparable maximum yield was found [40]. Other studies about algae biomass pretreatments are under way, in our laboratory, to increase the maximum hydrolysis yields.

### The hydrolytic efficiency of the *A. awamori*, *T. reesei*, and *A. cellulolyticus* enzyme pools and blends

Because the enzyme pool of *A. awamori* has mostly β-glucosidase activity in addition to endoglucanase activity and negligible cellobiohydrolase activity, a lower initial hydrolysis rate and a lower maximum hydrolysis yield were expected in comparison with the complete cellulases plus β-glucosidase preparations of *A. cellulolyticus* and the blend of *T. reesei* and *A. awamori* enzymes. In fact, we would expect a pattern of hydrolysis similar to the partially purified β-glucosidase, but with a higher initial rate and yield because it possesses endoglucanase activity and the partially purified β-glucosidase does not. However, a similar rate and hydrolytic efficiency were observed for the *A. awamori* enzyme preparation and the *A. awamori*-*T. reesei* blend in comparison with the *A. cellulolyticus* enzymes, indicating that exoglucanase (CBH) activity is not necessary for the hydrolysis of this microalgae biomass.

**Figure 3:**
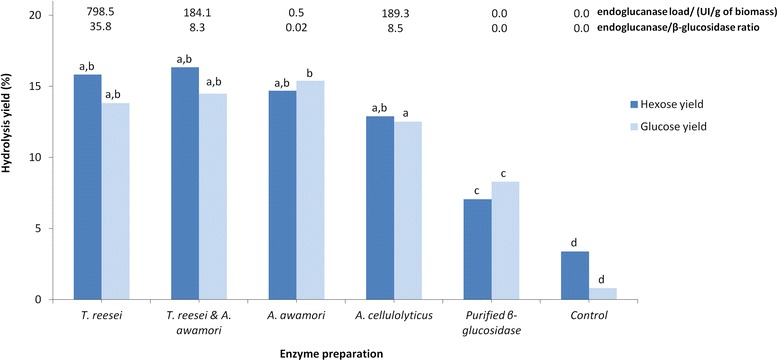
**The hydrolysis yield of** ***C. homosphaera*** **biomass after 3 h of enzymatic treatment.** *C. homosphaera* biomass (50 mg/mL) was incubated in 50 mM citrate buffer pH 4.8 at 50°C with the indicated enzyme preparation at a final β-glucosidase activity of 23.3 IU/g d.w. biomass load. Biomass without enzyme was incubated under the same conditions as the control. Letters a, b, c, and d indicate differences in the means at 0.05 level.

The use of the *A. awamori*-*A. cellulolyticus* enzyme blend resulted in a hydrolysis reaction medium with the highest β-glucosidase load (90.3 IU/g d.w. biomass) followed by the *A. cellulolyticus* enzymes (49.0 IU/g) and by both the *A. awamori* enzymes and the *T. reesei*-*A. awamori* enzyme blend (22.3 IU/g). The *T. reesei* enzymes showed the lowest β-glucosidase load (8.7 IU/g). With the exception of *A. cellulolyticus*, the initial hydrolysis rates and final yields correlated positively with the β-glucosidase. To understand further the importance of β-glucosidase activity, we conducted two sets of hydrolysis assays with *T. reesei*, *A. awamori*, *A. cellulolyticus*, and a blend of *A. awamori-T. reesei* enzyme preparations. In the first set, the assays were performed with the same β-glucosidase load for 3 h because more striking differences regarding the initial hydrolysis yields were observed within this time interval. A control experiment, with the same load of a partially purified β-glucosidase and devoid of endo- and exoglucanase activities, was conducted. Figure 3 shows that after 3 h of hydrolysis, no significant differences among the means of hydrolyzed hexose equivalents or glucose were observed for any enzyme preparation. However, the *A. cellulolyticus* enzymes showed lower mean values, mostly for glucose, which were significant when compared with the *A. awamori* enzyme, confirming the previous results shown in Figures 1 and 2, where despite having a higher β-glucosidase load, it showed a lower initial rate but a similar final hydrolysis yield.

**Figure 4:**
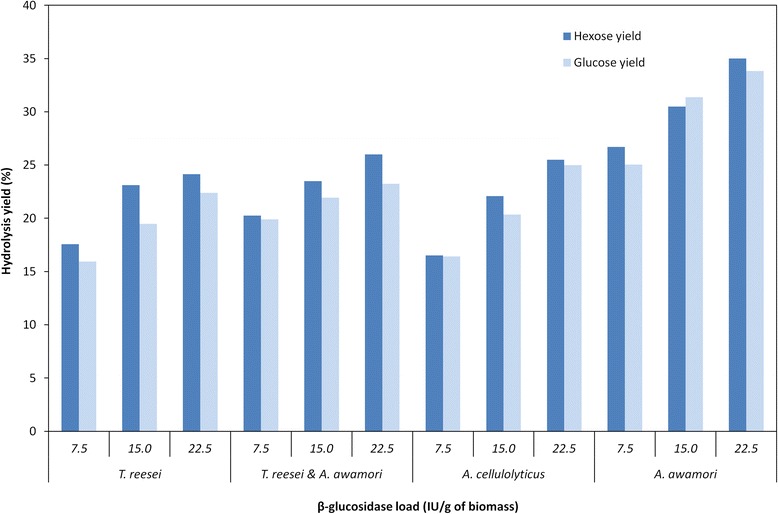
**The hydrolysis yield of** ***C. homosphaera*** **biomass after 36 h of enzymatic treatment.** *C. homosphaera* biomass (50 mg/mL) was incubated in 50 mM citrate buffer pH 4.8 at 50°C with 1.5 IU/g of endoglucanase load and β-glucosidase loads of 7.5, 15.0, and 22.5 IU/g biomass loads by adding partially purified β-glucosidase.

The hydrolysis results from the control experiments were surprisingly high, accounting for over 40% and 50% of the total hexose and glucose released, respectively (Figure 3). The data were even higher than that presented in Figures 1 and 2, which could be related to differences in the uneven manual grinding of the algae biomass, affecting the particle sizes as we observed in preliminary assays in our laboratory (data not shown). The *C. homosphaera* biomass also showed a small amount of biomass hydrolysis not caused by enzymatic action as previously found (Figures 1 and 2). The different enzyme preparations contained different endoglucanase loads (Figure 3), which might have played an important role in the hydrolysis results, although no correlation between the endoglucanase load and initial rates and final yields was found. For this reason, all enzyme preparations in the second set of assays were set to a fixed endoglucanase load (1.5 IU/g dry biomass) and increasing β-glucosidase loads as follows: 7.5, 15.0, and 22.5 IU/g dry biomass, which were achieved through purified β-glucosidase supplementation. Figure 4 shows that after 36 h of incubation, all enzymes showed an increase in the hydrolysis yield, especially when the β-glucosidase load increased from 7.5 to the 15 IU/g that was significant for all enzyme preparations. The *T. reesei* preparation showed no significant increase for the hydrolysis yield of reducing sugar when the β-glucosidase load increased from 15.0 to 22.5 IU/g; however, a significant increase in the hydrolysis yield of glucose was found. The same pattern was observed for the *A. cellulolyticus* preparation. However, no significant increase in the glucose hydrolysis yield was found for the *A. awamori* enzyme and the *A. awamori*-*T. reesei* blend; nevertheless, the reducing sugar yield increase was significant. An analysis among the groups showed that the hydrolysis yields for *A. awamori* enzyme preparations were the highest even when compared with experiments presenting a higher β-glucosidase load. Moreover, the *A. awamori* preparation was more responsive to the increase in the β-glucosidase load (Figure 4), indicating a unique catalytic feature for the *A. awamori* enzymes.

Data for the predicted kinetics parameters regarding the initial hydrolysis rates and maximum yields of each enzyme preparation with the three β-glucosidase load (Additional file 1: Figure S1 and Additional file 2: Figure S2) are shown in Table 4. The predicted maximum hydrolysis yield for all enzyme preparations followed the same pattern presented in Figure 4. The analysis within the group for the initial hydrolysis rates indicated that *T. reesei* and *A. cellulolyticus* preparations showed a significant increase when the β-glucosidase load was increased from 7.5 to 15 IU/g. A small increase was also found when the β-glucosidase load was increased from 15 to 22.5 IU/g, but the model failed to predict any significant difference in the initial rate values. *A. awamori* and *A. awamori*-*T. reesei* blend preparations showed no significant increase after the increment of the β-glucosidase load. An analysis among the groups showed that the *A. awamori* preparation showed the highest initial rates of reducing sugar release that were significant when compared with their counterparts, which showed no significant difference among them. The predicted initial rates for glucose release in the *A. awamori* preparation showed a large standard error and are not reliable, but the predicted means suggest higher initial rates when compared with the others.

**Table 4:** **The predicted kinetic parameters for** ***C. homosphaera*** **enzymatic hydrolysis yields at a 1.5 IU/g endoglucanase load and final β-glucosidase loads of 7.5, 15.0 and 22.5 IU/g**

**Enzyme preparation**	**β-Glucosidase load (IU/g dry biomass)**	**Predicted parameters**					
		**Hexose equivalents (SE)**			**Glucose (SE)**		
		***V***_0_ **(mg/g/h)**	***P***_max_ **(mg/g)**	***t*** **½ (h)**	***V***_0_ **(mg/g/h)**	***P***_max_ **(mg/g)**	***t*** **½ (h)**
*T. reesei*	7.5	48.2 (7.5)	203.3 (10.1)	9.5	59.1 (17.5)	184.8 (9.2)	9.3
	15.0	61.0 (16.9)	286.6 (19.1)	9.6	78.0 (17.8)	228.0 (8.9)	9.0
	22.5	93.4 (13.8)	275.1 (19.4)	6.8	91.9 (8.5)	253.5 (3.1)	7.8
*A. cellulolyticus*	7.5	53.2 (5.9)	176.0 (5.7)	6.5	59.4 (7.9)	190.7 (12.8)	6.4
	15.0	95.5 (13.1)	254.8 (11.6)	7.8	96.5 (10.6)	241.8 (12.2)	8.2
	22.5	98.3 (12.7)	271.1 (14.4)	5.6	129.9 (32.7)	292.2 (4.6)	8.2
*A. awamori*	7.5	165.3 (24.7)	273.8 (20,7)	1.5	239.4 (100.3)	249.4 (12.8)	1.0
	15.0	176.8 (29.6)	325.8 (14.0)	1.9	254.6 (181.1)	314.2 (15.1)	2.2
	22.5	158.5 (6.8)	452.5 (24.2)	3.6	246.9 (477.9)	329.2 (39.8)	2.4
*T. reesei* and *A. awamori*	7.5	63.9 (9.2)	226.0 (9.0)	4.2	71.6 (20.8)	226.3 (8.1)	8.2
	15.0	74.1 (15,2)	250.5 (12.4)	4.4	69.3 (17.3)	251.14 (12.0)	7.8
	22.5	95.6 (25.1)	290.6 (13.7)	5.3	88.2 (26.4)	248.7 (9.2)	12.4

The data were fitted into an exponential model as described in the ‘Materials and methods’ section, and the parameters were estimated as described in Table 3. The numbers in brackets account for the standard error of the estimation.

HPAEC-PAD analyses showed that the released monosaccharides consisted of only glucose and galactose for all hydrolysis conditions, i.e., 1.5 IU endoglucanase load/g (d.w.) biomass and 7.5, 15.0, and 22.5 IU β-glucosidase load/g (d.w.) biomass. The enzyme preparation of *A. awamori* showed the highest sugar hydrolysis yield, and the increment in the β-glucosidase load increased the amount of sugar that was hydrolyzed. These results are consistent with those presented in Figure 4.

Altogether, these results clearly show that for the same endoglucanase load, the hydrolysis yield of *C. homosphaera* biomass is positively affected by the β-glucosidase load. Moreover, even in the absence of *A. awamori* enzymes, the β-glucosidase load affects the initial rate of hydrolysis. This finding also shows that a very small endoglucanase load is needed to attain high hydrolysis yields within just 12 h of incubation. The higher rates and yields observed for *A. awamori* enzymes suggest that the enzyme prepared from this fungus have a higher ability to hydrolyze *C. homosphaera* biomass.

### *C. homosphaera* cell wall studies

**Figure 5:**
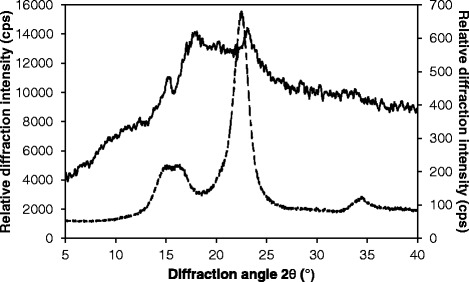
**Diffractograms of** ***C. homosphaera*** **biomass (continuous line, right axis) and Avicel Fluka (split line, left axis).** X-ray diffraction was performed using a Rigaku MiniFlex diffractometer and filtered copper Kα radiation.

The fact that the *A. awamori* enzymes, which are devoid of exoglucanase activity, hydrolyzed *C. homosphaera* biomass at high rates and that the β-glucosidase activity load influences the hydrolysis yields and sometimes the initial rate, raised a question regarding the nature of the microalgal cell wall glucan. Chlorophytes are known to be enriched with cellulose type I_α_, which is more susceptible to degradation than cellulose type I_β_, which is predominant in Charophyta and land plants [22,25,26,49]. Although the XRD of microalgae cellulose is generally similar to that of land plants, and except for some peaks at 14° and 16° that are not found in land plants [27], our results showed otherwise. Indeed, Figure 5 shows the X-ray diffractometry of *C. homosphaera* biomass and, for comparison, that of pure crystalline cellulose. The figure shows that, contrary to the data for crystalline cellulose, *C. homosphaera* biomass has a very low crystallinity, such that it was not possible to determine its degree of crystallinity. However, it was possible to identify a clear peak near 15°, which is typical for microalgae [27]. Remarkably, the XRD of *C. homosphaera* closely resembles that of the ball-milled XRD of microcrystalline cellulose after enzymatic hydrolysis, as found by Teixeira et al. [50], who also showed that cellobiohydrolase activity is not required to efficiently hydrolyze amorphous cellulose. These results suggest that the cell wall polysaccharides of *C. homosphaera* is mostly amorphous and, as such, *C. homosphaera* would be categorized as belonging to group 2, as established by Nicolai and Preston [17]. However, the low crystallinity may also be an artifact of biomass preparation; under stress, chlorophytes secrete a mucilaginous substance that was observed during the centrifugation process. In addition, the presence of algaenan in the cell wall may create structural changes and amorphous zones during the drying process, as suggested by Mihranyan [27]. However, regardless of whether the centrifugation and drying processes altered the cell wall polysaccharide structure, the dried *C. homosphaera* biomass showed such a low crystallinity that the enzyme mixtures devoid of exoglucanase activity were able to efficiently hydrolyze 40.5% of the algal glucan. In terms of biomass processing and enzyme use, this feature poses a great advantage for the biorefinery concept because it simplifies raw material processing and enzyme blend complexity, thereby reducing processing costs.

**Figure 6:**
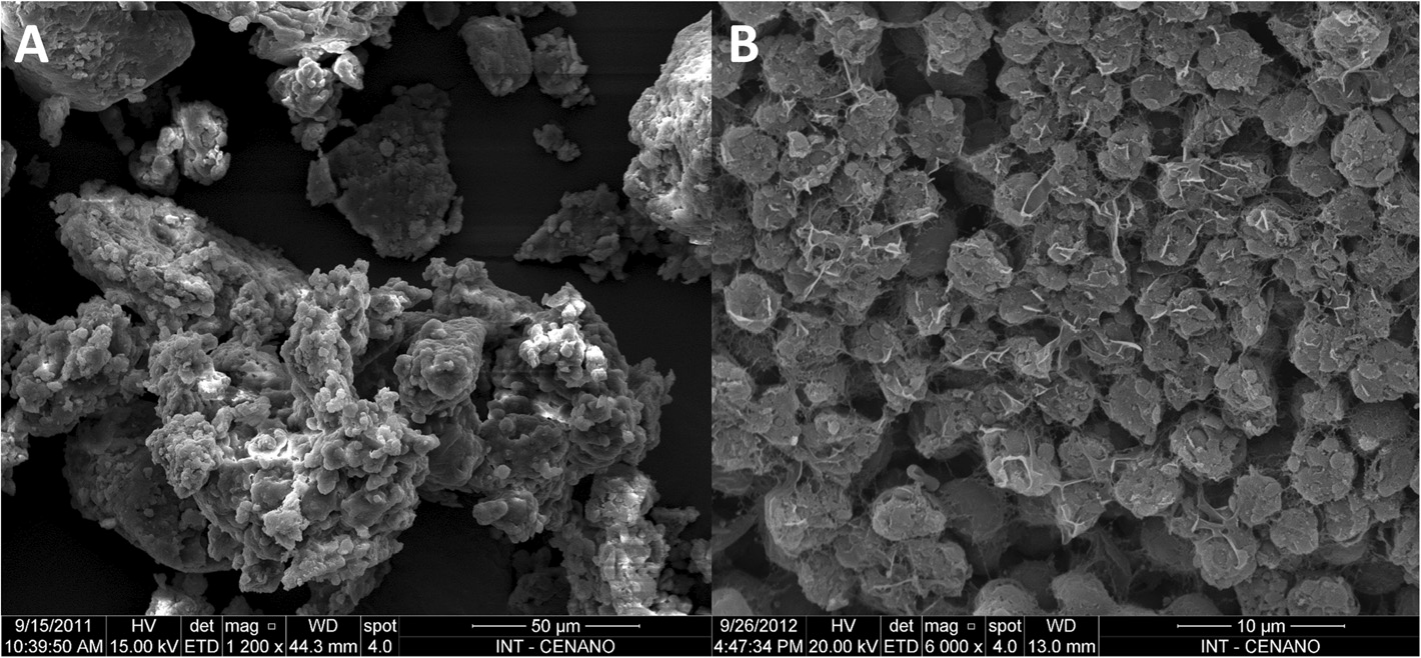
**Scanning electron microscopy of ground** ***C. homosphaera*** **biomass (A) before enzymatic hydrolysis and (B) after 48 h of enzymatic hydrolysis using the** ***A. awamori-T. reesei*** **blend.** Samples were adhered to carbon tape, sputter-coated with 28 nm gold using an Emitech/K550 model and observed via SEM as described in the Materials and methods section. Magnifications of × 1,200 (A) and × 6,000 (B) were chosen to show the action of the enzyme blend more clearly on *C. homosphaera* biomass. The scale bars represent 50 μm (A) and 10 μm (B).

Figure 6A shows the scanning electron microscopy of *C. homosphaera* cells and cell fragments that were dried and hand-milled, and Figure 6B shows the *C. homosphaera* cells after enzymatic hydrolysis when using the *A. awamori*-*T. reesei* enzyme blend. The images suggest that the enzyme action resulted in a more homogenous material and that an amorphous and sticky material that surrounded the undigested algae was removed by enzymatic hydrolysis.

## Conclusions

Enzyme mixtures of *A. awamori*, which have β-endoglucanase and β-glucosidase activities and are devoid of cellobiohydrolase activity, efficiently hydrolyze untreated *C. homosphaera* biomass, reaching 42.3% of the cell wall glucan because of its very low crystallinity, and the *A. awamori*-*A. cellulolyticus* enzyme blend attain even higher hydrolysis rates and yields of approximately 50%. This finding is most likely explained by the synergistic action of both enzyme preparations that allowed the hydrolysis of different polysaccharides that would be otherwise inaccessible. The β-glucosidase load was a determinant in the hydrolysis yield and in some cases for the initial hydrolysis rate of all the enzyme preparations.

## Materials and methods

### *C. homosphaera* cultivation

*C. homosphaera* cells were grown in inorganic W.C. medium [51] with continual aeration under white fluorescent light, with a 12-h photoperiod and a photosynthetic active radiation of 60 μmol/m^2^/s in average irradiance. The cells were cultivated in five carboys of 5 L each for 21 days and collected at the stationary phase of growth by centrifugation, then dried cold, ground manually on a mortar, and stored in the freezer until use, as previously described [52]. Under this growth condition, an average of 300 mg/L of dry weight biomass was obtained at the end of cultivation. Cell growth was followed by cell counting and by light scattering at 750 nm, to avoid the absorption of any cell chromophore, using a previously calibrated dry weight curve. Each growth phase was determined by plotting the logarithm of the cell concentration against the cultivation time.

### Fungal strains and enzyme preparations

Laboratory enzyme preparations were produced by *A. awamori* 2B.361 U2/1 and *T. reesei* Rut-C30 and deposited in the fungal culture collection of the National Institute of Quality Control in Health (INCQS 40259-40251, respectively) of the Oswaldo Cruz Foundation (http://www.incqs.fiocruz.br). Both fungi were propagated on potato dextrose agar (PDA) plates at 30°C for 7 days, until dense sporulation was observed. The spores were collected by adding 2 mL of sterilized distilled water to the plate, followed by gentle scraping. A standardized spore suspension presenting 10^7^ spores/mL in 20% (*v*/*v*) glycerol was maintained at −20°C. For enzyme production, *T. reesei* Rut C30 was cultivated in a liquid medium containing the following (in g/L): 30.0 lactose, 6.0 yeast extract, 0.3 urea, 0.6% (*v*/*v*) corn steep liquor, plus the following salts (in g/L): 1.4 (NH_4_)_2_SO_4_, 2.0KH_2_PO_4_, 0.3 CaCl_2_, and 0.3 MgSO_4_.7H_2_O, and the following trace elements (mg/L): 5.0 FeSO_4_.7H_2_O, 20 CoCl_2_, 1.6 MnSO_4_, and 1.4 ZnSO_4_, with an initial pH of 6.0. *A. awamori* was cultivated in a liquid medium containing the following (in g/L): 12.0 yeast extract, 30.0 wheat bran, 1.2 NaNO_3_, 3.0 KH_2_PO_4_, 6.0 K_2_HPO_4_, 0.2 MgSO_4_.7H_2_O, and 0.05 CaCl_2_, with an initial pH of 7.0 [53]. Enzyme production by both fungi took place in 1,000-mL Erlenmeyer flasks containing 300 mL of growth medium inoculated with 1% (*v*/*v*) of the spore suspension. The cultures were incubated in a rotary shaker (Innova 4340, New Brunswick, Edison, NJ, USA) at 30°C and 200 rpm. The commercial enzyme *Acremonium* cellulase from *A. cellulolyticus* was kindly provided by Meiji Seika Pharma Co., Japan.

*A. awamori* β-glucosidase was partially purified as follows. Fungal culture supernatant was concentrated by ultrafiltration using a 100-kDa membrane (Amicon Filtration System-Stirred Cells). A 10 mL volume of the retentate, which contained 227 mg of protein and a total β-glucosidase activity of 5672 IU, was subsequently fractionated by gel filtration on a Sephadex G-75 Column (3.0 × 62.5 cm) and pre-equilibrated with 50 mM sodium acetate buffer (pH 5.0) containing 0.15 M NaCl. The sample was eluted using a flow rate of 20 mL/h, and 5.0 mL aliquots were collected and screened for β-glucosidase activity and protein concentration by measuring the absorbance at 280 nm. Fractions presenting β-glucosidase activity were pooled and concentrated by ultrafiltration with a 30-kDa membrane. This preparation was shown to be free of FPase, CMCase, and xylanase activities. The protein concentration was also measured according to Bradford [54]. β-glucosidase preparations from gel filtration and ion-exchange chromatography were analyzed by SDS-PAGE, Native PAGE, and zymogram with an 8% polyacrylamide gel [55].

The following enzyme preparations were used to hydrolyze the *C. homosphaera* biomass: enzymes from *T. reesei* RUT-C30, *A. awamori*, *A. cellulolyticus*, and blends of *T. reesei*-*A. awamori* and *A. awamori*-*A. cellulolyticus*-excreted enzymes. The *T. reesei*-*A. awamori* blend was made by taking into account the FPase and β-glucosidase of each individual enzyme preparation, which were mixed in the correct proportion to achieve the final FPase: β-glucosidase activities ratio of 1:2. Moreover, hydrolysis experiments were performed with a purified *A. awamori* β-glucosidase preparation to evaluate the effect of this enzyme alone on the initial biomass hydrolysis rate. The aforementioned fungal enzymes were chosen because of their complementary activities as follows: (i) *A. awamori* 2B.361 U2/1, which produces high levels of glucoamylase and β-glucosidase, but lacks exoglucanase activity (8.4 IU/mL of β-glucosidase, 1.9 IU/mL of CMCase, and no FPase activity); (ii) *T. reesei* RUT C-30, which produces high levels of cellulases but low levels of β-glucosidase (1.4 IU/mL of β-glucosidase, 37.3 IU/mL of CMCase, and 1.2 IU/mL of FPase); (iii) *Acremonium* cellulase (Meiji Seika Co., Japan), which contains high CBH levels in addition to a complex set of biomass-hydrolyzing enzyme activities (29.2 IU/mL of β-glucosidase, 67.1 IU/mL of CMCase, and 4.3 IU/mL of FPase); and (iv) a blend of *A. awamori* and *T. reesei* after the concentration of each enzyme by ultrafiltration with a 30 kDa membrane (20.5 IU/mL of β-glucosidase, 69.5 IU/mL of CMCase, and 5.5 IU/mL of FPase).

### FPAse, CMCase, and β-glucosidase activity determinations

The activities of *A. awamori* 2B.361 U2/1 and *T. reesei* Rut-C30 laboratory enzymes and the *A. cellulolyticus* commercial enzyme were determined according to standard IUPAC procedures and expressed as international units (IU), as described by Ghose (1987) [56]. Several dilutions of the enzyme pools were prepared by diluting them with 50 mM citrate buffer, pH 4.8. Enzyme activities were estimated by incubating the diluted enzyme preparations with the relevant substrate in a water bath at 50°C for the time indicated. FPAse was determined by incubating exactly 6 cm^2^ of Whatman 1 filter paper for exactly 1 h. The reaction was stopped by adding 3,4-dinitrosalicylic acid (DNS) and centrifuging to remove paper debris; the reducing sugar content was then determined. The endo-β1,4-glucanase activity was determined by incubating a fresh 2% (*w*/*v*) carboxymethyl cellulose CMC 7 L2 solution in 50 mM citrate buffer, pH 4.8, for exactly 30 min, followed by the reducing sugar determination. The β-glucosidase activity was determined by incubating a fresh 15 mM cellobiose solution in 50 mM citrate buffer, pH 4.8, for exactly 30 min. The enzymatic reaction was stopped by incubating in a boiling water bath for 5 min; the glucose content was then determined. As controls, the reducing sugar contents of the paper and of the enzyme dilutions, in addition to the glucose contents of the enzyme dilutions, were determined. The enzyme activities were estimated using the calculations described in [56].

### *C. homosphaera* biomass enzymatic hydrolysis

The experiments were performed in 50-mL glass Erlenmeyer flasks that were stoppered with glass to minimize evaporation, with a 30-mL reaction mixture containing 50 mg/mL cell mass powder suspended in 50 mM citrate buffer, pH 4.8, and an enzyme load of 10 FPU (filter paper activity) units/g dry biomass. The measured β-glucosidase loads for the *T. reesei* and *A. cellulolyticus* preparations and the *A. awamori-A. cellulolyticus* blend were 8.7, 49.0, and 90.3 IU/g biomass, respectively. Because the *A. awamori* enzymes exhibited no FPAse, the enzyme load was equalized with the β-glucosidase load of the *T. reesei*-*A. awamori* blend, for 22.3 IU/g dry biomass. The mixtures were incubated at 50°C in a rotatory shaker (Innova 4340, New Brunswick, Edison, NJ, USA), and sampling was performed at 0, 2, 4, 6, 8, 12, 24, 28, 32, 36, and 48 h, followed by incubation for 5 min in a boiling water bath to halt enzyme action. Sugar analyses were performed in the supernatants of centrifuged samples.

To investigate the influence of the β-glucosidase activity on the initial enzymatic biomass hydrolysis and final yield, two sets of assays were designed. In the first, the biomass powder was incubated for 3 h under the same conditions as described above, with an enzyme load of 22.3 IU β-glucosidase/g dry biomass for all the enzymes. This time frame was chosen because the kinetics for the hydrolysis experiments using the evaluated enzyme preparations (*A. cellulolyticus*, *T. reesei*, *A. awamori*, and the *A. awamori*-*T. reesei* blend) with the same FPAse load but different β-glucosidase loads showed significant rate differences up to 3 h of hydrolysis, i.e., where the most β-glucosidase hydrolysis dependency was observed. For the controls, a sample of biomass powder was submitted to hydrolysis with partially purified *A. awamori* β-glucosidase and another biomass sample was incubated without any enzymes under the same conditions. As in the first set, the reaction medium of each enzyme preparation had a different endoglucanase load, and in the second set of the assay, each reaction medium of the evaluated enzyme preparations was set to have an endoglucanase load of 1.5 IU endoglucanase/g dry biomass. The β-glucosidase load was then adjusted by adding partially purified β-glucosidase to final load values of 7.5, 15.0, and 22.5 I.U/g dry biomass. All enzymatic assays were performed in triplicate.

### AHS determination

The AHS in *C. homosphaera* biomass was estimated on the basis of previously described procedures [57,58]. Soluble saccharides, lipids, and pigments were extracted by incubating 50 mg of biomass powder with 3 mL of 80% ethanol (*v*/*v*) for 30 min in a water bath at 70°C. The supernatant was discarded, and the procedure was repeated three times. The pellet was washed twice in 50 mM citrate buffer, pH 4.8, and resuspended in 0.9 mL of the same buffer; 0.1 mL of Novozyme amyloglucosidase AMG 300 L solution was then added to give a final enzyme activity load of 40 IU. The suspension was incubated for 24 h at 50°C, then centrifuged; the supernatant glucose content was then estimated. Sodium azide was added to prevent contamination.

### Nitrate determination

The nitrate remaining in the culture medium was determined by UV spectrophotometry as described in [59]. A 10 mL aliquot of cell suspension was withdrawn from culture cell flasks under sterile conditions. After determining the cell suspension turbidity at 750 nm, the cells were centrifuged and the clear supernatant was used to determine the nitrate content. For each 1 mL of supernatant, 20 μL of 1 mol/L HCl solution was added to prevent any interference from hydroxide and carbonate ions, and the absorbances at 220 and 275 nm were measured, assuring that the absorbance at 275 nm was less than 10% of the absorbance at 220 nm. The reading at 275 nm was used to discount the interference of organic materials that might be present in the sample. The net absorbance was calculated using the following equation: Net Abs = Abs(220) − 2 × Abs (275). The nitrate concentration was determined by means of an analytical curve of the net absorbance against the nitrate concentration, using a 20 μg/mL NaNO_3_ solution as a standard. All glassware was thoroughly rinsed with hot 1 mol/L HCl solution and distilled water before use, to eliminate any trace of carbonate and detergent.

### Sugar determination

The *C. homosphaera* total biomass sugar content was determined on the basis of the procedure described in [41]. A total of 4 mg of dry cell powder was incubated with 2 mL of 1 mol/L H_2_SO_4_ at 100°C for 6 h. The samples were then neutralized with BaCO_3_ and centrifuged, and the supernatants were used for total sugar determination by the phenol sulfuric method [60] with an analytical curve using a 0.2 mg/mL glucose solution as a standard. For the control, the same procedure was performed with pure glucose powder. The reducing sugar levels in hydrolysates were estimated by the 3,5-dinitrosalicylic method [61] by means of an analytical curve with a 2.0 mg/mL glucose solution as a standard. The glucose concentration was estimated using a YSI 2730 glucose analyzer (Yellow Springs Incorporated, Ohio, USA). The sugar composition was determined by HPAEC-PAD analysis.

### HPAEC-PAD analysis

A sugar composition determination was performed in an Ion Chromatography System 5000 (ICS-5000, Dionex Ltd., Canada) equipped with Chromeleon 6.8 (Dionex Ltd., Canada) software. The column system consisted of a CarboPac PA1 (4 × 50 mm, Thermo Scientific Ltd., USA) pre-column and a CarboPac PA1 (4 × 250 mm, Thermo Scientific Ltd., USA) analytical column. The chromatographic conditions were as follows: the samples were automatically injected at 15°C with an injection volume of 10 μL, a furnace temperature of 30°C, and a pressure of 1,050 to 1,150 psi. The detection was performed through a pulse amperometric detector specific for monosaccharides at 30°C. The mobile phase was a step gradient (0% to 85% to 0%) of 300 μmol/mL NaOH solution (reagent grade type I) and ultra-pure 0.2 μm filtered degassed water (with a resistivity of at least 18 MΩ) at a flow rate of 1.0 mL/min. The postcolumn solvent was a 400 μmoL/mL NaOH solution at a flow rate of 0.3 mL/min. The total running time was 50 min, and all solvents were kept under a N_2_ atmosphere.

Analytical curves (2.0 to 250.0 mg/L) were constructed for each monosaccharide to be determined using standards chosen based on previously reported *Chlorella* cell wall sugar compositions [42]. The sugar content was estimated by measuring the peak area.

### Curve fit analysis

The hydrolysis time curves were fitted in a double exponential curve, which was rejected when it did not converge or when the predicted parameters had *P* values greater than 0.05, which resulted in a large standard error. Under these circumstances, a mono-exponential model was used. Fittings were performed using a non-linear regression (Levengerg-Marquardt Algorithm) with SigmaPlot 10.0 software (Systat Software Inc., San Jose, CA, USA) for Windows. A fitting analysis was used to estimate the initial hydrolytic activity rates, the maximum hydrolysis yield, and the time needed to reach 50% hydrolysis for each enzymatic preparation (*t*½).

### Statistical analysis

The results obtained from the assays to test the dependency of biomass hydrolysis yields on β-glucosidase activity were analyzed by one-way ANOVA with MINITAB 15.0 software for Windows (Minitab Inc., PA, USA). The differences among the means were verified using the Fisher test. The means were considered to be significantly different when *P* ≤ 0.05.

### Cell wall X-ray diffractometry

The crystallinity of the *C. homosphaera* dried biomass was evaluated by X-ray diffraction with a Rigaku MiniFlex diffractometer and filtered copper Kα radiation (*λ* = 0.1542 nm) using a monochromator at a 30 KV voltage and 15 mA electric current, with a speed of approximately 2°/min and scanning at an angle (2*θ*) from 2° to 60°. For comparison, a diffractogram of cellulose microcrystalline (Avicel PH101, Sigma-Aldrich) was also performed. Each sample’s crystallinity index (CrI) was calculated according to the Segal method [62].

### SEM

Scanning electron microscopy (SEM-FEI/Inspect S50 model) was used to investigate the microalgae biomass morphology before and after 48 h of enzymatic treatment with the *A. awamori*-*T. reesei* enzyme blends. Samples were adhered to carbon tape, sputter-coated with 28 nm gold with an Emitech/K550 model and observed via SEM with an acceleration voltage of 20 KV and a working distance of approximately 19 mm. Several images were obtained from different areas of the samples (at least 20 images per sample) to guarantee the reproducibility of the results.

## Additional files

Additional file 1: Figure S1. The time course for the release of total reducing sugars from *C. homosphaera* biomass (50 mg d.w./mL) in 50 mM citrate buffer, pH 4.8, at 50°C. The biomass was hydrolyzed using enzyme preparations with a 1.5 IU/g endoglucanase load from *T. reesei* **(A)**, *A. cellulolyticus* **(B)**, *A. awamori* **(C)**, and a *T. reesei*-*A. awamori* blend **(D)** at final β-glucosidase loads of 7.5 (filled circle), 15.0 (empty circle), and 22.5 IU/g (triangles). The data were fitted into an exponential function as described in the Materials and methods section. Standard deviation was less than 10% of the mean value and bars were omitted for clarity. Additional file 2: Figure S2. A time course for the release of glucose from *C. homosphaera* biomass (50 mg d.w./mL) in 50 mM citrate buffer, pH 4.8, at 50°C. The biomass was hydrolyzed using the enzyme preparations with a 1.5 IU/g endoglucanase load from *T. reesei* **(A)**, *A. cellulolyticus* **(B)**, *A. awamori* **(C)**, and the *T. reesei*-*A. awamori* blend **(D)** at final β-glucosidase loads of 7.5 (filled circle), 15.0 (empty circle), and 22.5 IU/g (triangles). The data were fitted into an exponential function as described in the Materials and methods section. Standard deviation was less than 10% of the mean value and bars were omitted for clarity.

## Abbreviations

SEM: scanning electron microscopy
CrI: crystallinity index
FTIR: Fourier transform infrared spectroscopy
AHS: amyloglucosidase hydrolyzable starch
XRD: X-ray diffraction
FPAse: filter paper activity
FPU: filter paper unit
CMCase: endo-β-1,4 glucanase
INCQS: Instituto Nacional de Controle de Qualidade em Saúde
IU: international unit
IUPAC: International union of pure and applied Chemistry
TFA: trifluoracetic acid
EG: endoglucanase
CBH: cellobiohydrolase
HPAEC-PAD: high-performance anion exchange chromatography and pulse amperometric detection
