# Supplementary figures and images for: Untreated Chlorella homosphaera biomass allows for high rates of cell wall glucan enzymatic hydrolysis when using exoglucanase-free cellulases
Source: Biotechnol Biofuels. 2015 Feb 19;8:25. doi: 10.1186/s13068-015-0215-1 (PMC4356055; doi:10.1186/s13068-015-0215-1)

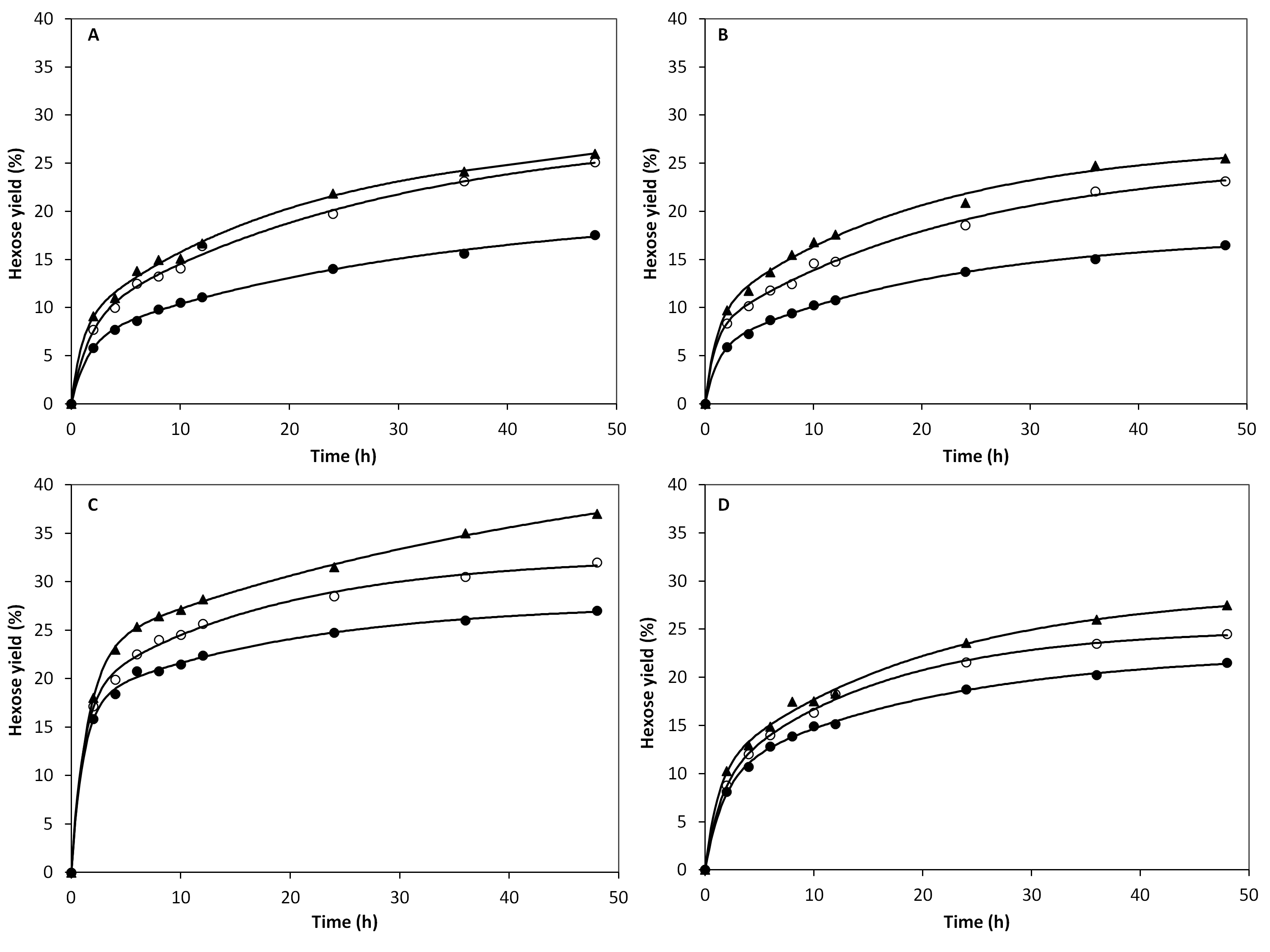

Supplement: Additional file 1: Figure S1. — The time course for the release of total reducing sugars from C. homosphaera biomass (50 mg d.w./mL) in 50 mM citrate buffer, pH 4.8, at 50°C. The biomass was hydrolyzed using enzyme preparations with a 1.5 IU/g endoglucanase load from T. reesei (A), A. cellulolyticus (B), A. awamori (C), and a T. reesei-A. awamori blend (D) at final β-glucosidase loads of 7.5 (filled circle), 15.0 (empty circle), and 22.5 IU/g (triangles). The data were fitted into an exponential function as described in the Materials and methods section. Standard deviation was less than 10% of the mean value and bars were omitted for clarity. [file 13068_2015_215_MOESM1_ESM.tiff]

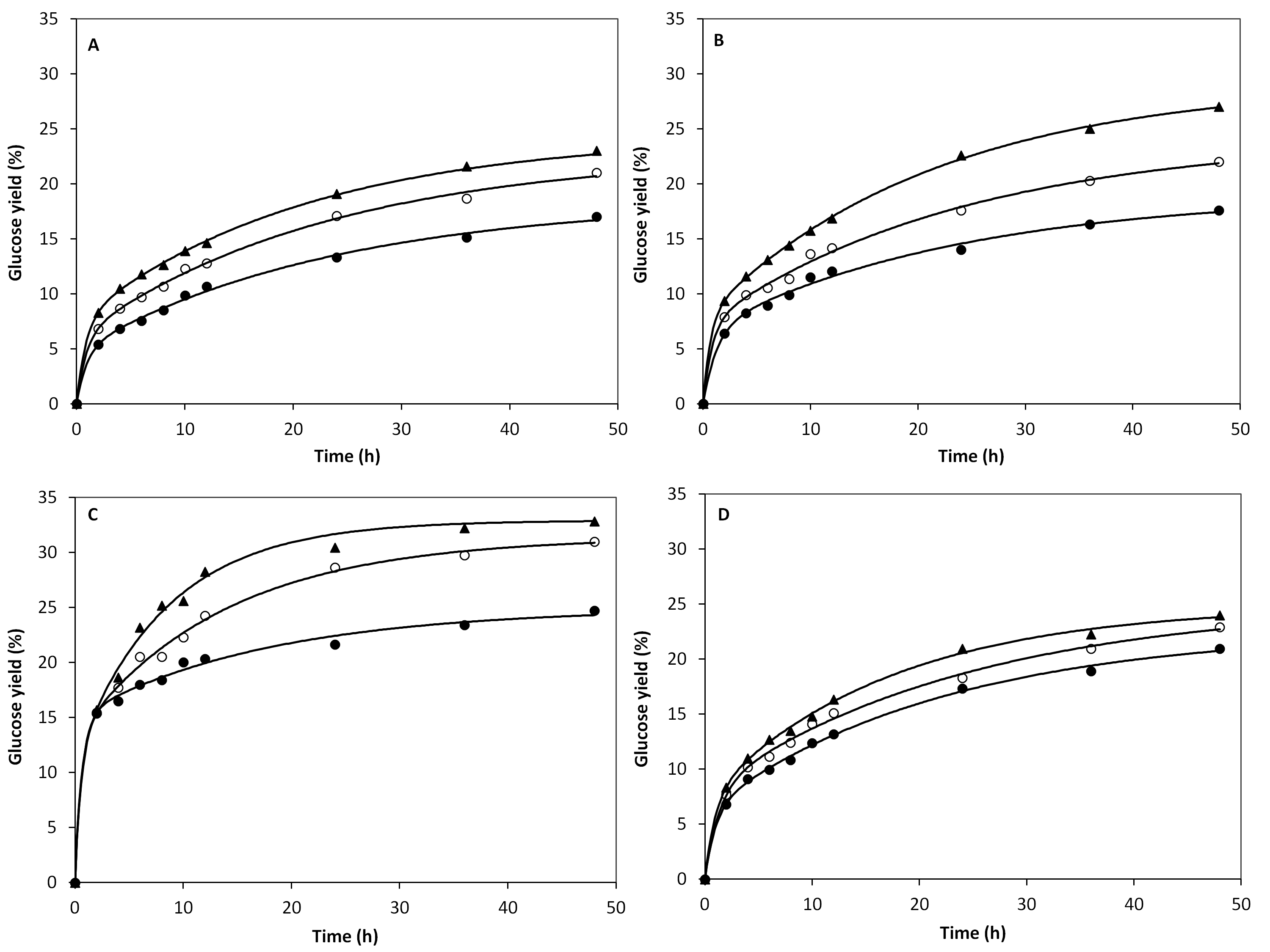

Supplement: Additional file 2: Figure S2. — A time course for the release of glucose from C. homosphaera biomass (50 mg d.w./mL) in 50 mM citrate buffer, pH 4.8, at 50°C. The biomass was hydrolyzed using the enzyme preparations with a 1.5 IU/g endoglucanase load from T. reesei (A), A. cellulolyticus (B), A. awamori (C), and the T. reesei-A. awamori blend (D) at final β-glucosidase loads of 7.5 (filled circle), 15.0 (empty circle), and 22.5 IU/g (triangles). The data were fitted into an exponential function as described in the Materials and methods section. Standard deviation was less than 10% of the mean value and bars were omitted for clarity. [file 13068_2015_215_MOESM2_ESM.tiff]
